# Effects of dry needling on muscle spasticity of the upper limb in a survivor of traumatic brain injury: a case report

**DOI:** 10.1186/s13256-022-03408-5

**Published:** 2022-06-14

**Authors:** Najmeh Sedighimehr, Saber Zafarshamspour, Mohammadhassan Sadeghi

**Affiliations:** 1 Physiotherapy Department, School of Rehabilitation, Shiraz University of Medical Sciences, Shiraz, Iran; 2 Student Research Committee, Faculty of Medicine, Shiraz University of Medical Sciences, Shiraz, Iran; 3 Consultant Neurosurgeon, Department of Surgery, Rafsanjan University of Medical Sciences, Rafsanjan, Kerman, Iran; 4 Medical Student, Zahedan University of Medical Science, Sistanbaluchestan, Iran

**Keywords:** Case report, Traumatic brain injury, Muscle spasticity, Dry needling

## Abstract

**Background:**

Survivors of moderate and severe traumatic brain injury typically present with spasticity, an upper motor neuron lesion associated with hyperexcitability of the stretch reflex due to disinhibition of cortical influences on spinal cord circuits and structural and functional changes in skeletal muscle. There is growing evidence supporting the effectiveness of dry needling in abating spasticity.

**Case presentation:**

The present case aims to quantify the effects of dry needling on upper limb spastic muscles in a survivor of severe traumatic brain injury in a 27-year-old Iranian man. The treated muscles were biceps brachii, brachialis, flexor digitorum superficialis and profundus, flexor carpi radialis, flexor carpi ulnaris, opponens pollicis, and adductor pollicis. Outcome measures were evaluated before and 1 hour after the intervention. Our results showed that the patient’s upper limb recovery stage and hand function improved one grade according to Brunnstrom recovery stages. Spasticity assessed using the Modified Modified Ashworth Scale in all movements showed one-grade abatement, except in the forearm pronator. Passive resistance force decreased in all movements except in forearm supination. Active range of motion and passive range of motion increased in all movements except in active and passive forearm supination. Hand dexterity improved in both affected and unaffected hands.

**Conclusions:**

Results shown that dry needling could be a favorable option for reducing spasticity.

## Introduction

Spasticity as one of the symptoms of upper motor neuron syndrome is characterized by a speed-dependent increase in muscle tone caused by excessive excitability of the stretching reflex [1]. Spasticity is mostly considered a neural lesion because the primary lesion leading to spasticity is located in the central nervous system. Recently, attention has been drawn to the structural and functional changes in skeletal muscle that occur secondary to spasticity. Studies have reported decreased mitochondrial volume fraction, appearance of intracellular amorphous material, reduction in muscle fiber length, and decrease in the number of serial sarcomeres within fibers of spastic muscles [2, 3]. Multiple therapeutic interventions including stretching, casting, splinting, pharmacologic treatment, botulinum toxin injection, and electrical stimulation of the muscles have been tried for abating spasticity that mostly involves the peripheral nerves and muscles [4–6]. Dry needling (DN) is a relatively new promising intervention that has been shown to control spasticity following stroke [7, 8]. It is assumed that DN not only decreases the spasticity by affecting contractile properties of spastic muscles [9] but also has modulatory effects on neural activity of the spinal circuit [10], supraspinal, and higher centers [11, 12].

Spasticity is a common condition among survivors of traumatic brain injury (TBI), and occurs in up to 20% of patients with moderate-to-severe TBI [13]. Up to 20% of patients with TBI develop an acute subdural hematoma [14], a condition that may be associated with contralateral spastic hemiplegia, a lesion whose physiopathology is similar to that of brain lesions of vascular origin [15]. When a lesion of the brainstem is involved, spasticity becomes more pronounced [16]. Spasticity occurs more commonly in upper limbs than lower limbs [17]. Upper limb function is severely compromised by spasticity owing to decreased range of motion, decreased voluntary strength, and increased joint stiffness [18, 19]. The aim of this case presentation is to evaluate the immediate effect of a DN session on a patient with left hemiplegia after TBI, who suffered from spasticity of the upper limb.

## Case presentation

The patient was a 27-year-old Iranian man surviving from severe TBI following a motor vehicle accident, with left hemiplegia and spasticity of the upper and lower limbs (lower limb spasticity less severe than upper limb), especially in the wrist flexors, pronator, and thumb adductor and flexor muscles. The patient had no history of any comorbid conditions such as cardiovascular disease, diabetes, or high blood pressure. He had started to walk independently and without support 8 months after injury. He had received several other treatments, including physiotherapy, exercise therapy, medication, and botulinum toxin injections to relieve spasticity of the upper limb, but still complained of spasticity and dysfunction in the left upper limb. We aimed to evaluate the immediate effect of a single session of DN on this patient with left hemiplegia after TBI, who suffered from spasticity of the upper limb. The study was approved by the research ethics committee of Shiraz School of Rehabilitation Sciences.

### Assessment

Outcome measures were evaluated before and 1 hour after the DN intervention.

- The upper limb recovery stage and hand function

The upper limb recovery stage and hand function were determined using a criterion defined by Brunnstrom [20] (Table 1), which has been introduced as a valid test for the assessment of patients with post-stroke hemiplegia [21].

b) Spasticity
  Spasticity was assessed by the Persian Modified Modified Ashworth Scale (MMAS) in the elbow, wrist, and finger flexors, forearm pronator, and thenar muscles. The MMAS grades the intensity of muscle spasticity on a 0–4 scale [22, 23] (Table 2). The patient was supine and the head was midline to prevent an asymmetric tonic reflex that may increase spasticity.
c) Passive resistance force
  Passive resistance force (PRF) was determined biomechanically by a handheld dynamometer (HHD) using the clinical method to assess spasticity with MMAS. The amount of Newtonian force (N) applied to the dynamometer sensitive pad by the evaluator against spasticity of the elbow, wrist, and finger flexors, forearm pronator, and thenar muscles to move them from flexion to extension and from pronation to supination at the end of the existing range of motion (ROM) and by gravity was measured. HHD is a reliable and valid tool for muscle evaluation in clinical settings [24].
d) Range of motion

**Table 1:** Brunnstrom recovery stages.

Stages	Description
Stage 1	Flaccidity; no movements of the limbs can be initiated
Stage 2	Spasticity begins to develop; the basic limb synergies or some of their components may be present Little or no active finger flexion can be performed.
Stage 3	Spasticity is marked; the basic limb synergies are performed voluntarily Mass grasp; hook grasp with no release; no voluntary finger extension
Stage 4	Spasticity begins to decrease; some movement combinations that deviated the synergies become available Small-range semi-voluntary finger extension
Stage 5	Spasticity is diminishing. More difficult movement combinations can be performed Voluntary mass extension of fingers
Stage 6	No spasticity; isolated joint movements can be performed normally Voluntary extension of fingers.

**Table 2:** Modified Modified Ashworth scale (MMAS)

0	No increase in muscle spasticity
1	Slight increase in muscle tone, manifested by a catch and release or by minimal resistance at the end of the ROM when the affected part(s) is moved in flexion or extension
2	Marked increase in muscle tone, manifested by a catch in the middle range and resistance throughout the remainder of the ROM, but affected part(s) easily moved
3	Considerable increase in muscle tone, passive movement difficult
4	Affected part(s) rigid in flexion or extension

A standard goniometer was used to measure the active ROM (AROM) and passive ROM (PROM).

- *Elbow extension* The patient was supine with the hand supinated. The axis of the goniometer was placed at the lateral epicondyle of the elbow, the stationary arm was parallel with the humerus, and the movement arm was parallel with the radius. Then, the assessor extended the patient’s elbow passively and recorded the end position of elbow extension as PROM. The same procedure was followed for the measurement of elbow extension AROM unless the patient was asked to extend voluntarily the elbow.
- *Wrist extension* The patient was in a sitting position next to the table, elbow flexed to 90°, and forearm in mid-position rested on the supporting surface. The axis of the goniometer was placed at the snuff box, the stationary arm was along the lateral radius, and the movement arm was aligned with the second metacarpal. Then, the assessor extended the patient’s wrist passively and recorded the end position of wrist extension as PROM. The same procedure was followed for the measurement of wrist extension AROM unless the patients were asked to extend voluntarily the wrist.
- *Forearm supination* The patient was seated with the shoulder adducted by their side, elbow flexed to 90°, and forearm in the neutral position to start. The axis of the goniometer was placed over the tip of the third digit. The stationary arm was parallel with the humerus, and the movement arm was aligned with the volar forearm surface. The assessor supinated the patient’s forearm passively and recorded the end position forearm supination as PROM. The same procedure was followed for the measurement of forearm supination AROM unless the patients were asked to supinate voluntarily the forearm.
- *Carpometacarpal (thumb) extension* The patient was in a sitting position next to the table, elbow flexed to 90°, and forearm in mid-position rested on the supporting surface. The axis of the goniometer was placed at the palmar aspect of the first carpometacarpal (CMC) joint, the stationary arm was along the ventral midline of the radius using the ventral surface of radial head and styloid for reference, and the movement arm was along the ventral midline of the first metacarpal. The assessor extended the patient’s thumb passively and recorded the end position of the thumb extension as PROM. The same procedure was followed for the measurement of thumb extension AROM unless the patients were asked to extend voluntarily the thumb.
- *Carpometacarpal (thumb) abduction* The patient was in sitting position next to the table, elbow flexed to 90°, and forearm in mid-position rested on the supporting surface. The axis of the goniometer was placed at the lateral aspect of the radial styloid process. The stationary arm was along the lateral midline of the second metacarpal. Reference center of the second MCP joint, and the movement arm was lateral midline of the first metacarpal. Reference center of first MCP joint. The assessor abducted the patient’s thumb passively and recorded the end position of thumb abduction as PROM. The same procedure was followed for the measurement of thumb abduction AROM unless the patients were asked to abduct voluntarily the thumb.
- *Box and Block Test* Reliable and valid Box and Block Test (BBT) was used to measure hand dexterity [25]. A test box, divided by a partition into two compartments of equal size consisting of 150 blocks, was placed in front of the patient on a standard-height table. The patient, sitting in a chair with a standard height, was instructed to remove the blocks one by one with the healthy hand and then with the injured hand, moving them from one compartment to another. The patient was encouraged to perform the test as quickly as possible. The number of blocks transferred to another test box chamber within 1 minute was recorded as BBT score.

### DN intervention

The patient received a DN session using disposable stainless steel needles (0.25 mm × 50 mm), which were inserted into the muscle through the skin. Treatment steps included hand washing, wearing disposable examination gloves, and cleansing the skin. The patient’s skin was prepared with an alcohol swab before intervention. Muscle positioning for DN was performed according to Dommerholt Second Edition [26]. The patient was lying down, arm away from the trunk, and forearm in the middle position. The target muscles received a DN using the fast-in and fast-out technique described by Hong [27]. Visual or tactile identification of the local twitch response (LTR) was considered a clinical sign of DN function because the patient’s main complaint was spasticity. Muscles treated were the elbow flexors (biceps brachii and brachialis), the wrist flexors (flexor carpi radialis, flexor carpi ulnaris, flexors digitorum superficialis, and profundus), and the thenar muscles (opponens pollicis, adductor pollicis).

- *Biceps brachii* The patient lay down supine with the arm slightly flexed. The muscle was grasped between the thumb and index and long fingers. Taut bands were identified. The muscle was needled from a lateral approach [26].
- *Brachialis* The patient lay down supine with the elbow relaxed and slightly flexed. The muscle was needled via flat palpation in the lateral aspect of the arm, distal two-thirds of the humerus. The needle was directed medially between the biceps and triceps brachii [26].
- *Flexors digitorum superficialis and profundus* The patient lay down in supine position with the forearm almost supinated. The muscles were needled with flat palpation; the needle was inserted perpendicular to the skin and directed toward the interosseous membrane [26].
- *Flexor carpi radialis (FCR)* The patient lay down in supine position with the forearm almost supinated. A point at the medial forearm, 4 cm below the point 1 cm medial to the midpoint of elbow crease, was needled for FCR.
- *Flexor carpi ulnaris (FCU)* The patient lay down in supine position with the forearm almost supinated. A point at the midpoint of the proximal third segment of a line from the medial epicondyle to the ulnar styloid process was needled for FCU.
- *Pronator teres (PT)* Pronator teres was needled at the proximal and medial forearm, 1 cm below the midpoint of elbow crease between the medial epicondyle and biceps brachii tendon.
- *Thenar muscles (opponens pollicis)* The needle was inserted at a point on the dorsum of the hand at the site of the proximal angle formed between the first and second metacarpal bones.
- *Thenar muscles (adductor pollicis)* The needle was inserted via the radial aspect of the first metacarpal (to avoid palmar fascia) toward the thenar eminence.

## Results

After a single session of DN, the patient’s upper limb recovery stage and hand function improved from 4 to 5 according to Brunnstrom recovery stages. Spasticity was assessed using MMAS in the elbow flexor, wrist, and finger flexors, and thumb flexor/adductor showed one-grade abatement in all except forearm pronator. PRF decreased in all movements that were measured except in forearm supination. Both AROM and PROM increased in all movements that were measured except in active and passive forearm supination. Also, hand dexterity, which was measured by BBT, improved in both affected and unaffected hands. Table 3 presents the pre- and post-intervention results.

**Table 3:** Pre- and post-treatment outcomes

Variables	Pre-intervention		Post-intervention			
Upper limb BRS	4		5			
Hand function BRS	4		5			
MMAS (elbow flexor)	1		0			
MMAS (forearm pronator)	3		3			
MMAS (wrist flexor)	2		1			
MMAS (thumb flexor)	3		2			
MMAS (thumb adductor)	4		3			
PRF(elbow flexor)	10		5			
PRF (forearm pronator)	30		30			
PRF(wrist flexor)	20		10			
PRF(thumb flexor)	10		7			
PRF (thumb adductor)	10		7			
AROM elbow extension	− 30		0			
AROM wrist extension	25		30			
AROM supination	15		15			
AROM thumb extension	0		5			
AROM thumb abduction	0		5			
PROM elbow extension	0		0			
PROM wrist extension	30		50			
PROM supination	20		20			
PROM thumb extension	30		70			
PROM thumb abduction	20		20			
Box and block test (boxes in 1 minute)	Affected side	Unaffected side		Affected side	Unaffected side	
	3	32		9	40	

*BSR* brunnstrom recovery stages,* MMAS* modified modified ashworth scale,* PRF* passive resistance force,* AROM* active range of motion,* PROM *passive range of motion

## Discussion

Spasticity is an uncompromising sequela of some neurologic diseases, including TBI, that is quite difficult to manage. Not only is this nettlesome for patients and negatively affects their function, especially upper limb function, but it is also a challenging problem for therapists working with these patients because spasticity can blunt the rehabilitation process. Therefore, controlling and mitigating spasticity is a therapeutic priority. Spasticity, a motor disorder associated with lesions of the central nervous system, is characterized by hyperexcitability of the stretch reflex due to disinhibition of cortical influences on spinal cord circuits resulting in a velocity-dependent increase in tonic stretch reflexes [28]. Spasticity is a neurological impairment resulting in structural changes and contractures in the muscles [29, 30] and, thus, increased stiffness of spastic muscle cells and tissue [2]. Dry needling is a common technique used by manual therapists to treat musculoskeletal disorders [31]. Recently, DN has emerged as a new option to treat muscle spasticity in neurological disorders such as post-stroke spasticity [32]. Some recent studies have provided evidence regarding the effectiveness of dry needles on abating spasticity [7, 8]. This case study aimed to evaluate the effects of DN in a patient suffering from upper limb spasticity post-TBI. Before DN intervention, the patient was able to combine some gross movement and deviate the synergies, and he had a small range of semi-voluntary finger extension, except thumb extension and abduction. After DN, he was able to combine more difficult movements as well as extend and abduct the thumb somewhat, in addition to voluntary extension of his fingers. This finding is similar to those reported in a previous study by Ansari *et al.* [8]. Significant improvement in hand functional outcomes after DN in patients with spasticity after stroke also has been proved neurophysiologically using H-reflex for α motor neuron excitability assessment [7]. In this case, spasticity after TBI showed one-grade abatement in almost all spastic muscles following DN, and consequently PRF against spastic muscles diminished. Our results are consistent with those of previous studies reported by Salom-Moreno *et al.* (2014) [33], Sánchez-Mila *et al.* (2018) [34] and Tavakol *et al.* (2019) [35], who also found a decrease in spasticity in the upper and lower extremities after a single session of deep dry needling. In contrast, Mendigutia-Gómez *et al.* (2016) found no change in spasticity in the shoulder muscles after dry needling [36]. In this patient, neither AROM nor PROM of supination changed after DN, which is in contrast with the results of Ansari *et al.* [8]. The lack of change in AROM and PROM may have been due to a contracture of pronators. However, the patient was able to cope with daily activities. The BBT as an indicator of manual dexterity improved after DN on the affected side, but also on the unaffected side. This may be explained as post-trial learning to some extent, as well as neural facilitation induced by DN [7], but this outcome needs further investigation. The main mechanism of DN-induced spasticity reduction is not fully understood, although there are several potential explanations. Since dry needles can reduce the stiffness of stretched bands [11], it can be assumed that dry needles can cause changes in spasticity by creating local tension release in the structure of shortened muscle fibers, as was shown in a patient with post-stroke spastic muscles [9]. Another potential explanation is related to the modulatory effects of DN on afferent and efferent neuron activity of the spinal circuit [10]. It seems that the local twitch responses elicited by DN can alter sensory spinal processing, then modulate α motor neuron excitability by modifying synaptic transmission and, therefore, decrease the excitability of spinal reflexes associated with muscle spasticity [37]. Furthermore, it is hypothesized that DN might also activate the noxious inhibitory control system by activating Aβ-fibres and Aδ fibres fibers sending afferent signals to the dorsolateral tracts of the spinal cord that could also activate the supraspinal and higher centers involved in pain processing [11, 12]. The activation of the noxious inhibitory control system following DN could have relaxing effects on the spastic muscles. The results of our study seem promising, though some potential limitations should be acknowledged. First, we collected short-term outcomes of a patient, while for accurate interpretation and generalization of the findings, it is necessary to evaluate larger samples of patients with spasticity, have a control group, and evaluate the outcomes with follow-up after treatment with DN. Second, it would be better to determine the effects of dry needling in combination with routine or other treatment options, as Cai *et al.* suggested that combining electroacupuncture with conventional treatment might be worthwhile in terms of muscle spasticity reduction, improvement of motor function, and return to activities of daily living [38]. Therefore, further research with a sufficient sample size, additional treatment sessions of DN, and combination with other treatment choices is suggested.

## Conclusions

The results of this case report suggest that application of a single session of dry needling on the upper limb spastic muscle was effective at decreasing muscle spasticity and improving passive resistance force, upper limb recovery stage, and hand function and ROM in individuals who had experienced traumatic brain injury. Future studies with larger sample sizes, more treatment sessions, long-term follow-ups, and inclusion of other treatment options are needed.

## Data Availability

All data generated or analyzed during this study are included in this published article.

## Abbreviations

TBI: Traumatic brain injury
LTR: Local twitch response
CMC: Carpometacarpal
HHD: Handheld dynamometer
DN: Dry needling

## References

[1] Mayer NH. Spasticity and other signs of the upper motor neuron syndrome. Spasticity: diagnosis and management: Demos Medical Publishing, New York; 2011. p. 17-31.

[2] Lieber RL, Steinman S, Barash IA, Chambers H. Structural and functional changes in spastic skeletal muscle. Muscle Nerve. 2004;29(5):615–27. 10.1002/mus.20059 15116365

[3] Olsson MC, Krüger M, Meyer LH, Ahnlund L, Gransberg L, Linke WA, *et al*. Fibre type-specific increase in passive muscle tension in spinal cord-injured subjects with spasticity. J Physiol. 2006;577(1):339–52. 10.1113/jphysiol.2006.116749 PMC2000690 16931550

[4] Rekand T. Clinical assessment and management of spasticity: a review. Acta Neurol Scand. 2010;122:62–6. 10.1111/j.1600-0404.2010.01378.x 20586738

[5] Bakheit AMO. The pharmacological management of post-stroke muscle spasticity. Drugs Aging. 2012;29(12):941–7. 10.1007/s40266-012-0034-z 23138834

[6] Graham LA. Management of spasticity revisited. Age Ageing. 2013;42(4):435–41. 10.1093/ageing/aft064 23775030

[7] Fakhari Z, Ansari NN, Naghdi S, Mansouri K, Radinmehr H. A single group, pretest–posttest clinical trial for the effects of dry needling on wrist flexors spasticity after stroke. NeuroRehabilitation. 2017;40(3):325–36. 10.3233/NRE-161420 28222554

[8] Ansari NN, Naghdi S, Fakhari Z, Radinmehr H, Hasson S. Dry needling for the treatment of poststroke muscle spasticity: a prospective case report. NeuroRehabilitation. 2015;36(1):61–5. 10.3233/NRE-141192 25547766

[9] Calvo S, Quintero I, Herrero P. Effects of dry needling (DNHS technique) on the contractile properties of spastic muscles in a patient with stroke: a case report. Int J Rehabil Res. 2016;39(4):372–6. 10.1097/MRR.0000000000000185 27362968

[10] Chou L-W, Kao M-J, Lin J-G. Probable mechanisms of needling therapies for myofascial pain control. Evid Based Complement Alternat Med. 2012;2012:1. 10.1155/2012/705327 PMC3549415 23346211

[11] Cagnie B, Dewitte V, Barbe T, Timmermans F, Delrue N, Meeus M. Physiologic effects of dry needling. Curr Pain Headache Rep. 2013;17(8):348. 10.1007/s11916-013-0348-5 23801002

[12] Hsieh Y-L, Hong C-Z, Liu S-Y, Chou L-W, Yang C-C. Acupuncture at distant myofascial trigger spots enhances endogenous opioids in rabbits: a possible mechanism for managing myofascial pain. Acupunct Med. 2016;34(4):302–9. 10.1136/acupmed-2015-011026 27143259

[13] Vivancos-Matellano F, Pascual-Pascual S, Nardi-Vilardaga J, Miquel-Rodríguez F, de Miguel-León I, Martínez-Garre M, *et al*. Guía del tratamiento integral de la espasticidad. Rev Neurol. 2007;45(6):365–75. 17899519

[14] Young RJ, Destian S. Imaging of traumatic intracranial hemorrhage. Neuroimaging Clin. 2002;12(2):189–204. 10.1016/s1052-5149(02)00003-5 12391631

[15] Lowe G, Twaddle S. The Scottish Intercollegiate Guidelines Network (SIGN): an update. Scott Med J. 2005;50(2):51–2. 10.1177/003693300505000202 15977513

[16] Brashear A. Spasticity: diagnosis and management: Springer Publishing Company; 2015.

[17] Lundström E, Smits A, Terént A, Borg J. Time-course and determinants of spasticity during the first six months following first-ever stroke. J Rehabil Med. 2010;42(4):296–301. 10.2340/16501977-0509 20461330

[18] Bhakta BB, Cozens JA, Chamberlain MA, Bamford JM. Impact of botulinum toxin type A on disability and career burden due to arm spasticity after stroke: a randomised double blind placebo controlled trial. J Neurol Neurosurg Psychiatry. 2000;69(2):217–21. 10.1136/jnnp.69.2.217 PMC1737061 10896696

[19] Meijer R, Wolswijk A, Eijsden Hv. Prevalence, impact and treatment of spasticity in nursing home patients with central nervous system disorders: a cross-sectional study. Disabil Rehabil. 2017;39(4):363–71. 10.3109/09638288.2016.1146351 26941031

[20] Liparulo L, Zhang Z, Panella M, Gu X, Fang Q. A novel fuzzy approach for automatic Brunnstrom stage classification using surface electromyography. Med Biol Eng Comput. 2017;55(8):1367–78. 10.1007/s11517-016-1597-3 27909939

[21] Naghdi S, Ansari NN, Mansouri K, Hasson S. A neurophysiological and clinical study of Brunnstrom recovery stages in the upper limb following stroke. Brain Inj. 2010;24(11):1372–8. 10.3109/02699052.2010.506860 20715900

[22] Naghdi S, Nakhostin Ansari N, Azarnia S, Kazemnejad A. Interrater reliability of the Modified Modified Ashworth Scale (MMAS) for patients with wrist flexor muscle spasticity. Physiother Theory Pract. 2008;24(5):372–9. 10.1080/09593980802278959 18821443

[23] Naghdi S, Ansari N, Mansouri K. Neurophysiological examination of the Modified Modified Ashworth Scale (MMAS) in patients with wrist flexor spasticity. Electromyogr Clin Neurophysiol. 2008;48:35–41. 18338533

[24] Stark T, Walker B, Phillips JK, Fejer R, Beck R. Hand-held dynamometry correlation with the gold standard isokinetic dynamometry: a systematic review. PM&R. 2011;3(5):472–9. 10.1016/j.pmrj.2010.10.025 21570036

[25] Chanubol R, Wongphaet P, Chavanich N, Chira-Adisai W, Kuptniratsaikul P, Jitpraphai C. Correlation between the action research arm test and the box and block test of upper extremity function in stroke patients. J Med Assoc Thai. 2012;95(4):590. 22612016

[26] Dommerholt J, Fernández-de-las-Peñas C. Trigger point dry needling e-book: an evidence and clinical-based approach: Elsevier Health Sciences; 2018.

[27] Hong C-Z. Lidocaine injection versus dry needling to myofascial trigger point. The importance of the local twitch response. Am J Phys Med Rehabil. 1994;73(4):256–63. 10.1097/00002060-199407000-00006 8043247

[28] Pandyan A, Gregoric M, Barnes M, Wood D, Wijck Fv, Burridge J, *et al*. Spasticity: clinical perceptions, neurological realities and meaningful measurement. Disabil Rehabil. 2005;27(1–2):2–6. 10.1080/09638280400014576 15799140

[29] Gracies JM. Pathophysiology of spastic paresis. II: emergence of muscle overactivity. Muscle Nerve. 2005;31(5):552–71. 10.1002/mus.20285 15714511

[30] Lorentzen J, Grey MJ, Crone C, Mazevet D, Biering-Sørensen F, Nielsen JB. Distinguishing active from passive components of ankle plantar flexor stiffness in stroke, spinal cord injury and multiple sclerosis. Clin Neurophysiol. 2010;121(11):1939–51. 10.1016/j.clinph.2010.02.167 20457538

[31] Kalichman L, Vulfsons S. Dry needling in the management of musculoskeletal pain. J Am Board Fam Med. 2010;23(5):640–6. 10.3122/jabfm.2010.05.090296 20823359

[32] Uttam M. To explore the literature on the effects of dry needling on muscle spasticity: a scoping review. Res Rev J Neurosci. 2015;5:40–2.

[33] Salom-Moreno J, Sánchez-Mila Z, Ortega-Santiago R, Palacios-Ceña M, Truyol-Domínguez S, Fernández-de-las-Peñas C. Changes in spasticity, widespread pressure pain sensitivity, and baropodometry after the application of dry needling in patients who have had a stroke: a randomized controlled trial. J Manipulative Physiol Ther. 2014;37(8):569–79. 10.1016/j.jmpt.2014.06.003 25199825

[34] Sánchez-Mila Z, Salom-Moreno J, Fernández-de-Las-Peñas C. Effects of dry needling on post-stroke spasticity, motor function and stability limits: a randomised clinical trial. Acupunct Med. 2018;36(6):358–66. 10.1136/acupmed-2017-011568 29986902

[35] Tavakol Z, Shariat A, Ghannadi S, Noormohammadpour P, Honarpishe R, Cleland JA, *et al*. The effect of dry needling on upper and lower limb spasticity in a patient with a brain tumor. Acupunct Med. 2019;37(2):133–5. 10.1177/0964528419830401 30845810

[36] Mendigutia-Gómez A, Martín-Hernández C, Salom-Moreno J, Fernández-de-Las-Peñas C. Effect of dry needling on spasticity, shoulder range of motion, and pressure pain sensitivity in patients with stroke: a crossover study. J Manipulative Physiol Ther. 2016;39(5):348–58. 10.1016/j.jmpt.2016.04.006 27167369

[37] Liu Q-G, Liu L, Huang Q-M, Nguyen T-T, Ma Y-T, Zhao J-M. Decreased spontaneous electrical activity and acetylcholine at myofascial trigger spots after dry needling treatment: a pilot study. Evid Based Complement Alternat Med. 2017;2017:1. 10.1155/2017/3938191 PMC5448056 28592980

[38] Cai Y, Zhang CS, Liu S, Wen Z, Zhang AL, Guo X, *et al*. Electroacupuncture for poststroke spasticity: a systematic review and meta-analysis. Arch Phys Med Rehabil. 2017;98(12):2578–89. 10.1016/j.apmr.2017.03.023 28455191

